# On spillovers in economic evaluations: definition, mapping review and research agenda

**DOI:** 10.1007/s10198-023-01658-8

**Published:** 2024-01-23

**Authors:** María J. Mendoza-Jiménez, Job van Exel, Werner Brouwer

**Affiliations:** 1 Erasmus School of Health Policy & Management (ESHPM), Erasmus University Rotterdam, Rotterdam, The Netherlands; 2 Erasmus Centre for Health Economics Rotterdam (EsCHER), Erasmus University Rotterdam, Rotterdam, The Netherlands; 3 Facultad de Ciencias Sociales y Humanísticas, Escuela Superior Politécnica del Litoral (ESPOL), Guayaquil, Ecuador

**Keywords:** Spillovers, Spillover effects, Spillover costs, Economic evaluation, Definition, D60, D61, D62, I00

## Abstract

An important issue in economic evaluations is determining whether all relevant impacts are considered, given the perspective chosen for the analysis. Acknowledging that patients are not isolated individuals has important implications in this context. Increasingly, the term “spillovers” is used to label consequences of health interventions on others. However, a clear definition of spillovers is lacking, and as a result, the scope of the concept remains unclear. In this study, we aim to clarify the concept of spillovers by proposing a definition applicable in health economic evaluations. To illustrate the implications of this definition, we highlight the diversity of potential spillovers through an expanded impact inventory and conduct a mapping review that outlines the evidence base for the different types of spillovers. In the context of economic evaluations of health interventions, we define spillovers as *all impacts from an intervention on all parties or entities other than the users of the intervention under evaluation*. This definition encompasses a broader range of potential costs and effects, beyond informal caregivers and family members. The expanded impact inventory enables a systematic approach to identifying broader impacts of health interventions. The mapping review shows that the relevance of different types of spillovers is context-specific. Some spillovers are regularly included in economic evaluations, although not always recognised as such, while others are not. A consistent use of the term “spillovers”, improved measurement of these costs and effects, and increased transparency in reporting them are still necessary. To that end, we propose a research agenda.

## Introduction

Economic evaluations are regularly performed to inform decision makers about the incremental costs and (health) benefits of new health interventions, such as curative treatments, use of medical devices, immunisation programmes, and other initiatives that aim to improve the health of a particular patient group or the wider population. An important issue in this context is the scope that economic evaluations should take for assessing the costs and benefits of health interventions. While this issue is often discussed in terms of the perspective adopted by an economic evaluation [1], most notably a broad societal perspective or a narrower healthcare perspective, it also concerns which costs and benefits should be included in the evaluation [2]. That is, given the perspective that is adopted, do economic evaluations include all relevant elements of value to adequately inform the decision-making process?

This question concerns, for instance, which outcome is considered to be relevant (e.g., health or well-being) and which costs need to be included in the evaluation (e.g., future costs or caregiving time costs). However, it also concerns *for whom* such effects might occur or *on whom* such costs might fall. This is important because if all relevant costs and effects are not included in an economic evaluation, it will provide only partial insight into the value of the intervention. Such incomplete information can lead to suboptimal decisions about whether to fund particular interventions from the available budget for healthcare.

Furthermore, it is increasingly acknowledged that patients are not isolated individuals; their health problems and their healthcare consumption may also affect other people, in different ways. Such impacts on others should not be ignored when *relevant* for the analysis. This perspective aligns with the “rule of reason”^1^ presented by the First US Panel on Cost-Effectiveness in Health and Medicine [3], guiding the selection of impacts to be included in the analysis.

Two prominent groups that are affected by the health of patients and their healthcare consumption are informal caregivers and family members. In 1996, the First US Panel already recommended including time costs related to informal care in economic evaluations and encouraged analysts to ‘think broadly’ about health effects on significant others, such as informal caregivers and family members [3]. Including the health effects on these groups may be relevant when adopting either a healthcare or a societal perspective in economic evaluations [4, 5], whereas including the time costs of informal caregivers or broader impacts may be relevant only when taking a societal perspective [6].

In this context, the term “spillovers” (or “spillover effects”) has been used to highlight that patient health, healthcare consumption, and changes therein may have a substantial impact on others beyond the patient [4, 7]. Although, in the context of economic evaluations, the term “spillovers” has been used in relation to costs and especially effects on informal caregivers and family members, some studies have used this term more broadly to include the impacts (often costs) on wider groups (e.g., colleagues or society)—for instance, by considering productivity impacts, health impacts on future lives or costs beyond the healthcare sector [8–11]. Moreover, multiple health technology assessment (HTA) agencies presently recommend including (some of) these broader impacts, in line with the perspectives adopted in their country [9, 12]. While this illustrates that the inclusion of costs and (health) effects for those other than patients is increasing, as is the evidence documenting (the relevance of) such costs and effects, there is a lack of clarity about what exactly is meant by “spillovers” of health interventions. Muir and Keim-Malpass [13], for instance, have recently argued that an improved conceptualisation of “spillovers” would be helpful.

We emphasise here that whether or not an impact is labelled as a spillover is not directly relevant in determining whether it should be included in an economic evaluation. Aligning with the perspective chosen for the evaluation, all relevant impacts should be included. Conceptual clarity regarding intervention spillovers will allow a comprehensive approach to this topic, help identification of potentially relevant impacts on others, and facilitate the development of a coherent body of knowledge to assess the relevance of broader impacts of health interventions. This paper therefore aims to clarify and define the concept of spillovers in the context of economic evaluations of health interventions. Given the broad scope of the definition, we discuss its practical implications for economic evaluation studies, and we propose a systematic way to identify potentially relevant spillovers in the form of an expanded impact inventory. We then outline the evidence base for costs and effects that are identified as spillovers, with an emphasis on those that are less commonly associated with the term. Finally, we suggest several areas for future research in this field.

## Spillovers: definition, implications, and identification

According to Grosse and colleagues [7], the term “spillover effects” was introduced in the context of health economic evaluations by Basu and Meltzer [4].^2^ Using a family utility function, they showed that the medical consumption of one family member can have direct and indirect effects on the welfare of the other family members, which they labelled “spillovers”. While the use of the term was new in this context, the assertion that the health and healthcare consumption of one person could affect the health, wealth, and well-being of others was not new. For instance, the broader costs of providing informal care [3, 14] as well as the health and well-being effects in informal caregivers and other family members due to illness and treatment of patients had been investigated before [15]. In line with Basu and Meltzer, most other research on spillover effects has focused on informal caregivers^3^ and family members, especially in terms of health effects [13, 16]. However, while one might expect certain effects to be largest in these groups, there is no a priori reason why spillovers should be confined to health impacts on informal caregivers and family members. For instance, health benefits for unvaccinated individuals (e.g., through herd immunity) are well-established population-level benefits of national immunisation programmes (NIPs) against infectious diseases [17]. Such effects may also be considered to be spillovers.

Moreover, spillovers need not be restricted to health effects alone. Indeed, previous authors have already broadened the concept of spillovers to include costs, both within the social networks of patients and beyond [1, 7]. The term “spillovers” has also been used to refer to costs and effects of healthcare interventions occurring in sectors other than the healthcare sector [8, 9], which indicates the potentially broad scope of the concept. Again, this assertion is not new, as analysts adopting a societal perspective have long been encouraged to include all costs and effects, regardless of where or on whom they occur [3]. Consequently, it has been advocated that costs typically not borne by the individuals undergoing an intervention, such as productivity costs [18] and costs in other sectors like education [19], should be included in economic evaluations that adopt a societal perspective. These costs have not always been labelled as spillovers, but can certainly be seen as such, as they affect people other than the patients themselves. In line with the general aim of capturing all relevant impacts in an economic evaluation, such broader impacts should not be ignored when they are deemed relevant for decisions based on the perspective adopted for the economic evaluation. When adopting a healthcare perspective, in which the aim is often assumed to be health maximisation from a given healthcare budget, health effects on others than patients may be considered relevant. In addition, costs incurred by others that impact the healthcare budget may also be considered relevant. When adopting a societal perspective, all costs and (health) effects on others are, in principle, relevant.

Despite the multiple uses of the term “spillovers” in the context of health economic evaluations, to our knowledge, there is currently no definition that coherently connects the term with the full range of elements it is associated with. Some authors have attempted to provide conceptual clarity about spillovers. For instance, Muir and Keim-Malpass [13] defined “spillover effects” as the “health impacts and costs that extend beyond a health intervention or program’s targeted recipient (the patient) to unintentionally impact other recipients either in a positive or negative way”. They highlighted opportunities to expand the scope of “spillovers” to include impacts beyond the patient’s social network, at different levels of the healthcare system and on other sectors of the economy. Francetic et al. [20] introduced a taxonomy for identifying and measuring “spillover effects” in healthcare policy implementation. Based on a review and previous conceptualisations of spillovers in the contexts of behavioural spillovers and impact evaluations [21, 22], they described “spillover effects” as those concerning “non-targeted units” and/or “non-intended effects”. The targeted unit was defined as the “unit that the intervention explicitly aims to affect and which should experience a change in outcomes as a result”, and the intended effects as the “outcomes which are expected to change by whoever designs an intervention, as a result of the implementation of the intervention itself.”

Three differences between these previous conceptualisations are worth noting. First, Muir and Keim-Malpass seem to exclude non-health impacts from the definition of “spillover effects”, whereas Francetic et al. do not. Second, Muir and Keim-Malpass explicitly acknowledge costs as a type of “spillover effects”, whereas Francetic et al. do not. This may be explained by the fact that the focus of the latter was the evaluation of policy intervention programmes, and not economic evaluations per se. Third, Muir and Keim-Malpass seem to confine spillovers to unintentional impacts on others (i.e., “diagonal spillover effects” according to Francetic et al.), whereas Francetic et al. consider that spillovers may also be intended effects on others (i.e., “between-units spillover effects”) or unintended effects on the targeted unit (i.e., “within-unit spillover effects”).

In this paper, we argue that whether or not impacts (costs or effects) are intended is not relevant for the definition of spillovers, but only whether they concern parties or entities other than the user of the intervention. In other words, we propose that it is crucial and central for a coherent definition of spillovers in health economic evaluations that spillovers are defined relative to the units undergoing the health intervention to be evaluated, i.e., they spill over from those units to others. Thus, we propose the following definition:

In the context of economic evaluations of health interventions, *spillovers* are all impacts from an intervention on all parties or entities other than the users of the intervention under evaluation.

According to this definition, none of the impacts of an intervention on the users of the healthcare product or service under evaluation are considered spillovers, whereas all impacts of the intervention on others are considered spillovers. In healthcare, the “users” or the units undergoing the intervention are typically patients, but they can also include households or families (e.g., in the case of family therapy), larger groups (e.g., an age cohort or a community), or even populations (e.g., universal mass vaccination).^4^ “All parties or entities other than the user” refers to those affected by spillovers. These may be people, ranging from close relatives (e.g., partners, children, or parents) to all taxpayers or citizens (e.g., when health interventions are collectively paid for) but also organisations (e.g., productivity losses), sectors (e.g., healthcare or education), and broader entities (e.g., the environment). Obviously, “all impacts” has a far-reaching scope; it includes costs as well as effects, which may be intended or unintended impacts, positive or negative, and may result directly from the intervention (i.e., from changes in users’ current health or care needs) or indirectly (i.e., from changes in users’ longevity and future care needs or opportunity costs elsewhere in the system).

Unlike the definitions provided by Muir and Keim-Malpass [13] and Francetic et al. [20], we have not conditioned spillovers on the “targeted unit” of an intervention but on the “user” of that intervention. This is deliberate, as defining who is targeted by an intervention may not be straightforward, thus complicating the distinction between spillovers and impacts on targeted units or main effects. Consider, for example, an intervention that involves training parents to help improve the mental health of their child. Following our proposed definition, the parents would be the “users” of the intervention and experience the main effect, while the impact of the intervention on their child’s mental health would be an intervention spillover. Moreover, although an NIP may be targeted at people over 65 years old, not all of them may choose or be able to receive the vaccination. In our definition, only those who receive the vaccination are considered intervention “users”, enabling a clear distinction between the main effect and spillovers. Consequently, health gains in unvaccinated individuals would qualify as a spillover.

Given the broad scope of our definition compared to the definitions that have implicitly been applied in the literature, some implications require emphasis. In a general sense, our definition includes costs and effects that are commonly labelled as spillovers. For instance, health effects on informal caregivers and non-caregiving family members of patients (i.e., those “caring for” and “caring about” them) qualify as spillovers, and both should still be included in economic evaluations whenever they are deemed influential, in line with the “rule of reason” of the First US Panel [3]. The same applies to the costs of informal care. However, our definition also classifies as spillovers some items that are commonly included in economic evaluations but not typically labelled as spillovers. For example, if the costs of an intervention are collectively financed and thus (largely) borne by others (i.e., through premiums or taxes), these costs would qualify as spillovers, as they are not (fully) paid by the users of the intervention. Out-of-pocket payments by users would not qualify as spillovers but may in fact be seen as reducing related spillovers, by lowering the costs borne by others. Moreover, our definition includes costs and effects that may be less frequently included in economic evaluations but are nonetheless spillovers, such as costs in other public sectors like education or justice, as well as productivity losses for colleagues of care users [23, 24]. Lastly, by defining spillovers relative to intervention users, the definition implies that impacts stemming from a treated user to another treated user are not intervention spillovers. Consider a scenario where partner A and partner B have received the COVID-19 vaccine. If partner A provides care to partner B due to vaccine side effects such as flu-like symptoms, the care-related impacts on partner A are not labelled as intervention spillovers, according to our definition, because partner A is also a user of the intervention. Nevertheless, these impacts could be significant and relevant to be included in an economic evaluation.

Analogous to adopting a societal perspective, our definition of spillovers highlights the relevance of thinking broadly about the consequences of patients’ health and healthcare interventions for others. At the same time, given the broad array of potential spillovers, it is important to approach the identification of relevant spillovers systematically, as also advocated by Francetic et al. [20]. Here we highlight two important steps to be applied in the context of any particular intervention.

First, it must be determined *who* are the users of an intervention, which may target patients, families, residents in certain geographical areas, or even populations. If all family members in a household use an intervention, no spillovers occur within the family. Obviously in this case, items such as the costs of informal care or health effects for informal caregivers (i.e., “caregiving effects”) living in the household and non-caregiving household members (i.e., “family effects”) can still be included in an economic evaluation, but in this case, they will not qualify as spillovers as per our definition. Moreover, if the spillover of a health intervention is not conditional on the number of users (unlike, for instance, herd immunity for vaccination), our definition implies that the size of the group experiencing spillovers decreases as the number of users increases.

Secondly, it must be determined *who or what, other than the users*, might be affected by the health intervention, and *which impacts* the health intervention might have on these others. Depending on the perspective adopted, the relevant spillovers may involve health impacts on others, broader impacts (e.g., well-being or care-related quality of life) on others, and costs within or outside the healthcare sector, at an individual level or higher (e.g., criminal justice or environmental costs). Table 1 illustrates the use of the proposed definition by listing potential spillovers from examples of interventions provided by healthcare professionals that are aimed at improving patient outcomes.

**Table 1:** Identifying potential spillovers of health interventions; illustrative examples

Intervention	Type	User	Potential spillovers	
		Who?	Who?	Which?
Pharmaceutical	Drug or vaccine	Patient	Caregivers, family, others (e.g., other relatives, employer, society)	Health-related quality of life, well-being, informal care time costs, productivity costs, environmental costs
Psychological therapy	Group therapy sessions	Patient and family	Others (e.g., other relatives, employer, society)	Health-related quality of life, education sector costs, criminal justice costs
Physical therapy	Individual therapy sessions	Patient	Caregivers, family, others (e.g., other relatives, employer, society)	Health-related quality of life, care-related quality of life, productivity costs
	Group training sessions	Informal caregivers	Patient, family, others (e.g., other relatives, employer, society)	Health-related quality of life, well-being, productivity costs

If economic evaluations aim to fully inform decision makers about the value of an intervention, then, in principle, all relevant spillover costs and effects need to be included in the evaluation. Providing general guidance for identifying relevant spillovers of interventions is challenging, as the nature and magnitude of these spillovers might depend on the specific evaluation context. Nevertheless, an established generic framework (i.e., not disease-specific) may be helpful for enumerating potentially relevant spillovers in a systematic manner. Here we use the impact inventory template proposed by the Second US Panel [25], which is increasingly being used in published CEAs since 2016 [9] and has already been adapted to consider broader consequences in specific contexts, such as human papillomavirus [26], Hodgkin’s lymphoma (ibid) and the COVID-19 pandemic [27].^5^ This inventory of health and non-health impacts promotes the consideration of broader consequences of interventions before quantifying and valuing them [27, 28].

To allow for a systematic consideration of spillovers, we have expanded the original inventory with an extra column to distinguish between impacts on intervention users and others (see Table 2). The rows in column 3 were populated by analysing each impact (i.e., each item in column 2) and determining whether it is a spillover, or whether an equivalent spillover, as per our definition, might be applicable (see table notes). The impacts in column 3 are not exhaustive, but rather highlight the potential diversity of spillovers of health interventions; future studies may well identify additional spillover categories. We relied on the original labels of the inventory to create the categories in column 3. Two further adjustments were needed; the references to “patients” were changed to “users”, and “health outcomes” was shortened to “outcomes” to acknowledge the broader range of impacts that our proposed definition includes. Columns 4 and 5 indicate whether the impact is relevant for the healthcare and societal perspective, respectively.

## Spillovers in practice

The broad scope of our definition, as presented in the previous section does not imply that all spillover costs and effects must be included in all evaluation studies. In practice, the relevance of each spillover will be context-specific, and how to quantify this *relevance* is an empirical question. To provide some guidance for the identification of potentially relevant spillovers in different contexts, this section outlines the empirical evidence related to the categories of spillovers displayed in the expanded impact inventory (see Table 2, column 3). This overview is based on a mapping review [29, 29, 29], taking a broad approach to the extensive literature on spillovers in economic evaluations of health interventions. Appendix 1 describes the search strategy applied. We limited the scope of our review to evaluations of the common forms of patient-level health interventions, which also leads to the largest set of spillovers given our definition.

For consistency, we henceforth use “spillover effects” to refer to impacts on others quantified in natural units or valued in monetary or non-monetary terms, such as the health-related quality of life (HRQoL). “Spillover costs” refers to impacts on others in terms of resource uses valued in monetary terms, such as productivity costs.

**Table 2:**
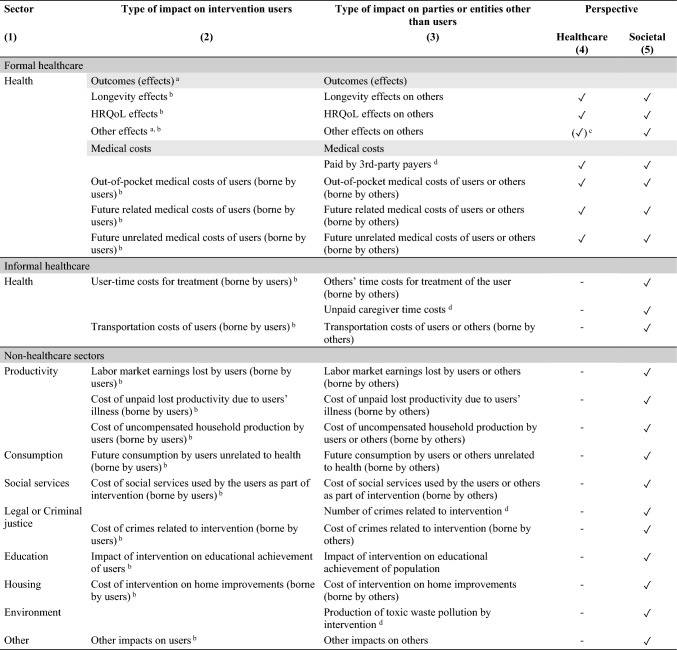
Expanded impact inventory

This is an expanded version of the impact inventory by the Second US Panel [25] that distinguishes between impacts on intervention users and impacts on others

^a^Compared to the original label, the word “health” was removed to acknowledge outcomes broader than health, such as well-being or care-related QoL

^b^Impact was differentiated into impact on (or costs borne by) intervention users and impact on (or costs borne by) others

^c^Relevance to the healthcare perspective depends on the specific effect under consideration

^d^Impact that fully classifies as a spillover

### Effects

#### Health outcomes

In the literature, “spillover effects” is most frequently used to describe health impacts on individuals other than the patients undergoing the intervention. This may include changes in longevity [30] but mostly relates to changes in HRQoL. The health of others may be affected by changes in the patient’s health, a patient’s death or by the intervention itself, for example, through changes in care needs, emotional and relational mechanisms, or biological transmission channels [17, 31, 32].

Incorporating the health effects of interventions on others has become part of the standard set of recommended practices since the First US Panel [33], and these are relevant when taking a societal or healthcare perspective. Currently, HTA bodies around the world are increasingly developing guidelines for incorporating HRQoL effects on others in reference cases for economic evaluations (e.g., England [34], France [35], Ireland [36], and the Netherlands [37]) or explicitly recommending their exclusion (e.g., Australia [38] and New Zealand [39]). As with patient health effects, the most commonly used HRQoL measures are the EQ-5D [40] and the SF-6D [41].

A considerable amount of the empirical literature presents evidence of spillovers of *diseases* on the HRQoL of others, mostly informal caregivers and family members [42, 43]. Mental and emotional health dimensions appear to be especially affected in these groups. For example, Landfeldt et al. [44] reported lower mental health scores among caregivers of patients with Duchenne muscular dystrophy as compared to general population values. Similar findings have been obtained among caregivers or family members of seriously ill individuals [45], cancer patients [46], and meningitis survivors [47]. In addition, bereaved family members may also experience lower HRQoL relative to the general population [48].

In the context of health *interventions*, multiple reviews have assessed whether “health spillovers”, i.e., health impacts on others, are considered in published economic evaluations [49–52]. These reviews highlight that only a minority (4–23%) of the studies included the HRQoL of others, even in disease areas where the effects are expected to be substantial, such as Alzheimer’s disease or paediatric diseases. In the context of public health interventions involving criminality, HRQoL impacts on victims could also be regarded as spillover effects. For instance, Ramponi et al. considered victims’ QALY losses in an economic evaluation of three interventions to reduce alcohol consumption among offenders on probation, based on a randomised controlled trial (RCT) [53].

When the HRQoL outcomes of others are considered and enough information to identify health spillovers is provided, multiple scenarios can unfold. Evaluation studies have identified differences across alternatives that are not statistically significant or negligible in size [54, 55], or significant even when patients’ health does not change [56]. Other studies have reported health spillover effects in favour of the intervention for several treatments, such as meningitis vaccinations [57], rotavirus vaccinations [58], cognitive stimulation therapy [59], treatment for spinal muscular atrophy [60], home palliative care programmes [61], and medication for Alzheimer’s disease [62, 63].

More recently, Scope et al. reviewed 40 cost-utility analyses (CUAs) that included informal caregivers’ or family members’ HRQoL [64]. They outlined current trends related to health spillovers of interventions. The most frequently evaluated treatment was vaccinations (15 of 40 studies). Only 13 studies obtained caregivers’ or family members’ health utilities from clinical trials, mostly interventions for dementia, while other studies retrieved these utilities from secondary sources, e.g., from different disease contexts or by making assumptions about the magnitude of spillover effects [57, 65]. In addition, the authors noted variations in the number of affected others, whether QALYs for bereaved relatives were included, and whether HRQoL was measured as utilities or disutilities.^6^ Although fewer than half of the studies (15 out of 40) reported the impact of including others’ HRQoL measurements on the calculation of incremental cost-effectiveness ratios (ICER), when they did, the ratios generally decreased (in 10 out of 15 studies). This pattern may also be due to selective inclusion or reporting.

Altogether, despite the growing attention being paid to health spillovers, especially on informal caregivers and family members, they are infrequently included in economic evaluations. When they are, the sources of evidence and the methods used vary considerably. Moreover, it often remains unclear what the magnitude of the effect is (e.g., because it is aggregated with patient outcomes) or how it is distributed across family relationships (e.g., spouses, parents, children)[66]. While this review was not intended to provide detailed guidance on how to measure, value and aggregate health spillovers (including whether or not they should receive the same weight as patient health), it is worth noting the growing research contributing to the quantification and incorporation of health spillovers in economic evaluations [5, 6, 47, 67, 68].

#### Other effects

Concerns have been voiced regarding the suitability of common HRQoL measures, such as the EQ-5D or SF-6D, to capture (all) relevant spillover effects. Given their focus on physical health, they may not adequately measure all relevant dimensions [51, 69, 70]. For example, depressive symptoms in informal caregivers were not always found to result in lower HRQoL scores than for the general population [71, 72], suggesting the absence of additional important elements. HRQoL measures might not be sensitive enough to capture changes in the general quality of life (QoL) dimensions that are most relevant to those caring for or about the patient, such as emotional health, quality of relationships, and fulfilment from caregiving [52, 64, 73]. In response, alternative outcome measures have been developed, including (i) measures of QoL specifically designed for caregivers and (ii) measures of general QoL or well-being [16]. These types of outcomes broaden the scope of economic evaluations to cover outcomes beyond health, which was the main benefit measure in the original impact inventory. This broader scope may not always be compatible with the assumed goal of the decision makers that are informed by economic evaluations.

Regarding *caregiver-specific quality of life*, it is well documented that providing informal care can affect a broad range of life domains, positively and negatively, and that these effects are not all captured by common HRQoL measures [6, 31, 73–75]. These effects may occur not only through changes in the care recipients’ health, but also via other mechanisms such as the care recipients’ engagement with healthcare services or the conditions in which these services are provided [31]. Different measures have been developed to capture these effects, focusing on aspects such as the caregiver’s burden, care-related QoL, management and coping, emotional and mental health, and psychosocial impacts [76, 77]. However, these measures were generally developed for evaluating interventions aimed at caregivers and are therefore mostly not suited for inclusion in economic evaluations of interventions for patients [78–81]. For example, different preference-based multi-attribute measures of care-related QoL have been developed, such as the Adult Social Care Outcomes Toolkit for Carers (ASCOT-Carer) [82], the Care-related Quality of Life (CarerQol) instrument [75], and the Carer Experience Scale (CES) [83]. Wittenberg et al. [42] identified seven studies reporting caregiver outcomes using the CarerQol or the CES, but none in the context of an intervention. A relevant study to highlight is the pre-post evaluation of an information and communication technology training for visually impaired adults by Patty et al. [84]. CarerQol measurements of caregivers were reported, but no significant differences were found across time periods. It is worth noting that the outcomes obtained from these measures cannot be added to patient QALYs [6], as they measure different concepts. Caregiver-specific QoL outcomes could, however, be considered alongside health outcomes in patients in multi-criteria decision analyses, but we did not come across any such studies in our mapping review.

Regarding *general quality of life* (or well-being), different measures have been developed that may be more suitable when interventions do not only, or primarily, aim to improve health [85–89]. Such well-being measures may also be relevant for assessing spillovers and facilitate the aggregation of effects on both patients and others. Several multi-attribute well-being instruments are available, including the 10-item Well-being instrument (WiX) [90, 91], ICECAP-A [92] and the QoL instrument developed by the World Health Organization (WHOQOL-BREF) [93] for the general population. For older adults, the ICECAP-O [94] and the WOOP [95] have been developed. Recently, the EQ Health and Wellbeing measure (EQ-HWB) was announced and continues under development [96]. Although these instruments should facilitate the measurement and inclusion of well-being spillovers in economic evaluations, so far they have only been used in a limited number of cross-sectional studies for identifying spillovers of *diseases*. For example, the shortened version of the WHOQOL-BREF has been used to identify spillovers of diseases among family caregivers of people with schizophrenia in Spain [97] and people with intellectual disabilities in Taiwan [98], as well as changes in well-being over time among caregivers of people with alcoholism in Germany [99]. Although the number of evaluation studies reporting well-being patient measurements is growing [89], we did not identify any randomised study measuring well-being outcomes for individuals other than the patient or modelling these impacts.

### Costs

An analogy between the broad scope of spillovers and the broad scope of the societal perspective is especially salient when exploring spillover costs. In empirical studies, the societal perspective is mostly conceptualised as “all costs irrespective of the payer” [100]. It follows that exploring diverse spillover costs aligns with the implementation of the societal perspective.

In this subsection, we present the findings in the literature for a selection of spillover costs as examples of lesser-known spillovers that can result from considering spillovers systematically, as described in “Spillovers: definition, implications, and identification” (*who or what, other than the user*, and *which impacts*). Although caregiving time costs are receiving increased attention, other spillovers affecting informal caregivers are often overlooked, such as medical and productivity costs, which are outlined below. Moreover, interventions may lead to costs borne by different parties or entities, also outside the household, such as employers and ultimately society as whole. To illustrate this further, we highlight spillover costs in the education sector and environmental costs. Appendix 2 complements the following overview with the empirical evidence related to the other spillover costs listed in the inventory: future medical costs, unpaid caregiver time costs, transportation costs, costs in the legal or criminal justice sector, and other spillover costs. Findings related to spillovers outside the formal healthcare sector are particularly relevant for analysts involved in regulatory frameworks that recommend the societal perspective as the preferred choice for reference cases (e.g., the Netherlands [37], France [35], Sweden [101], Finland [102], and Thailand [103]) or that allow consideration of the societal perspective in non-reference case analyses (e.g., United States [104], Canada [105], and Brazil [106]).

#### Medical costs

Medical costs of patients falling on others than the user of an intervention (e.g., collectively financed through health insurance) are spillover costs according to our definition. These costs are commonly included in economic evaluations [9, 28], although not labelled as spillover costs. Here we focus on the medical costs of others than the intervention users, either out-of-pocket or collectively financed. These costs may be a direct consequence of the intervention or an indirect consequence (e.g., when care is provided). Previous studies have, for example, reported significant associations between patient health status and the healthcare utilisation of informal caregivers in cases of dementia [107], attention deficit hyperactivity disorder (ADHD) [108], and cancer [46]. For mothers of children with ADHD, healthcare use was mostly associated with ambulatory mental health services and psychotropic medication [108]. Similarly, Schmitz and Stroka [109] reported a higher intake of antidepressants and tranquilisers among employed individuals with caregiving responsibilities compared to those without such responsibilities. These costs may reduce the health spillovers related to caregiving, which underscores the relevance of considering medical spillover costs in economic evaluations and, in turn, the consistency of measuring the full impact of interventions.

Nevertheless, medical spillover costs are rarely reported. A review of 51 cost-of-illness studies (COIs) did not document any measurement of healthcare costs for individuals other than the patients [110]. The consideration of medical spillover costs in economic evaluations is also rare. Krol et al. [50] reviewed 100 CUAs of interventions for patients with dementia, of which only three quantified medical costs of caregivers, and Lavelle et al. [52] reviewed 142 CUAs of paediatric patient interventions, of which only two included medical costs of caregiving parents. Differences across treatment arms were not identifiable, as costs were not disaggregated.

#### Productivity costs

Productivity costs can be defined as “the costs associated with production loss and replacement due to illness, disability and death of productive persons, both paid and unpaid” [111]. In the impact inventory, this is the first category of costs in the non-healthcare sector (see Table 2). It can be an influential cost category in economic evaluations taking a societal perspective [9, 112]. Available evidence largely relates to changes in patient productivity, which constitute spillovers whenever the associated costs fall on others, such as employers (i.e., wages) or society (e.g., lost added value, increased costs of social security). Patient productivity costs, during and after treatment, are frequently included in economic evaluations of health interventions [9, 18], though not systematically. Changes in the productivity of others than the intervention users have received much less attention.

Although care-related *time* inputs are included in the informal caregiver time costs, interventions may impact productivity through mechanisms other than the time spent on caring for a patient. For instance, productivity may be affected by mental distress caused by the patients’ health status or through long-term employment effects after the caregiving tasks have ended or the disease has been avoided. Productivity may also be affected by bereavement [113]. Distress and bereavement can affect not only informal caregivers, but also non-caregiving relatives, friends, and others in the patient’s social network. Similarly, spillovers may occur in a patient’s colleagues, due to so-called multiplier effects [23, 24], in which the absenteeism or presenteeism of a patient also leads to productivity losses for colleagues. Moreover, changes in productivity may be further valued as gains (or losses) concerning larger groups in certain disease contexts, e.g., population-level productivity gains via herd effects of vaccination against infectious diseases [17].

Some HTA guidelines recognise the relevance of impaired productivity (presenteeism) among employed informal caregivers (e.g., Canadian guidelines [105]) but not presenteeism while engaging in other activities such as leisure and unpaid work, while these also represent societal costs. Productivity costs in patients and others can, for instance, be measured using standardised instruments like the Work Productivity and Activity Impairment instrument [114] or the iMTA productivity costs questionnaire [115]. Such instruments generally retrieve the number of hours or days missed from work (absenteeism) and the number of hours lost while working due to impaired productivity (presenteeism).

Several studies have reported productivity-related spillovers associated with *diseases*. For instance, Goren et al. [46] reported higher absenteeism and presenteeism due to own health issues among caregivers of cancer patients, compared to non-caregivers. Long-term consequences for caregivers have been documented as well, e.g., a lower probability of returning to work and wage penalties among female caregivers of elderly adults [116], as well as early retirement among caregivers of veterans with long-term injuries [117]. Such findings suggest that caregiver time costs may not fully reflect the impacts of health interventions on the productivity of others. Regarding productivity-related spillovers of *interventions*, some economic evaluations include both productivity costs and caregiver time costs [65, 118]. However, we did not come across studies that provide enough detail to clearly disentangle these costs, and double counting should be avoided. To address this issue, Landfeldt et al. [119] have proposed a standardised questionnaire for the measurement and valuation of (paid and unpaid) informal caregivers’ time and productivity costs as separate mutually exclusive cost types for incorporation in economic evaluations.

Similar to patients [112], intervention spillovers due to productivity losses in unpaid work other than informal care also remain underexplored when they concern others, including secondary caregivers and non-caregivers. Exceptions include model-based evaluations in which spillover outcomes are of primary interest, such as interventions aiming to improve children’s outcomes by improving parenting behaviours [120].

#### Education

Health interventions may also impact resource use in the education sector, and some HTA guidelines explicitly acknowledge the potential relevance of these spillovers (e.g., the Dutch and Canadian guidelines [105, 121]). These costs may relate to the educational needs of health intervention users, which represent spillovers, as these are typically borne by others. However, resources from the education sector may also be utilised by individuals beyond the users of the intervention. For instance, young caregivers of veterans with long-term injuries have reported cutting back on school due to caregiving responsibilities [117], which may lead to higher educational costs. Negative impacts on educational outcomes have also been found for older siblings due to the health condition of a younger sibling [122, 123].

Pokhilenko et al. [124] identified 24 intersectoral costs and benefits related to the education sector that are relevant in the context of mental health interventions, e.g., home education costs, absenteeism, and reduced school engagement. Furthermore, in a systematic review of COIs and economic evaluations of interventions in mental health, psychosocial, and educational interventions, Pokhilenko et al. [19] extracted information from 49 studies that measured and valued costs in the education sector. The proportion of these costs in relation to the total costs of the intervention ranged from 0 to 67%. Economic evaluations that incorporate education spillover costs generally measure the resource use by children or adolescents who undergo an intervention, such as in the context of alcohol-use prevention [125]. Some evaluations include the use of educational services by younger individuals even when they are not the users of the intervention. For instance, Kuklinski et al. [120] conducted a cost–benefit analysis of a randomised home-visiting intervention for caregivers of children aged 0–5, focusing on the prevention of child maltreatment. Similarly, Gardner et al. [126] conducted a cost-effectiveness analysis to evaluate a parenting programme designed to prevent disruptive behaviour in children over a period of 25 years. Both evaluations estimated long-term cost savings in terms of the children’s utilisation of education sector resources, such as special education placement and counselling.

#### Environment

The climate footprint attributable to the healthcare sector amounts to approximately 4.4% of global net emissions [127]. Furthermore, assessments of the carbon footprint of healthcare services are increasingly available in the literature [128–130]. In the context of health economic evaluations, Desterbecq and Tubeuf [131] document studies that have incorporated environmental costs as an additional cost component. These costs represent spillovers, according to our definition.

For instance, De Preux and Rizmie [132] compared in-centre versus home haemodialysis of patients with chronic kidney failure. Using secondary data, they included carbon emissions related to the treatments. The costs represented less than 1% of the total costs for both groups, and their inclusion did not significantly alter the ICER. Similarly, Marsh et al. [133] extended an economic model to include environmental outcomes in an evaluation of antidiabetic regimens with and without basal insulin therapy. Building on this study, Hensher [134] valued the carbon footprint estimates by attaching shadow prices from the literature, which changed the ICER only slightly (around 3%). Waste reduction benefits have also been estimated, for instance, in the context of thermostable vaccines delivered through micro array patch [135].

It is likely that these spillovers will be included more often in the coming years, especially as public policy aligns more strictly with sustainability goals. For example, a recent measure requires government suppliers to develop carbon reduction plans in the United Kingdom [136]. It thus seems worthwhile to better understand the circumstances under which environmental spillovers can be impactful.

## Discussion and a research agenda

The aim of this study was to clarify and define the concept of spillovers in the context of economic evaluations of health interventions. We have defined spillovers as *all impacts from an intervention on all parties or entities other than the users of the intervention under evaluation*. While the scope of the definition is broad, the relevance of spillovers is context-specific. We have proposed a systematic way to identify potentially relevant spillovers by expanding the impact inventory template developed by the Second US Panel [25]. Guided by this framework, we then presented a mapping review of the different spillover types that have been explored in evaluation studies to date.

While spillovers of health interventions have typically been associated with impacts on informal caregivers and family members, our review shows that a broader range of consequences may be relevant and extend beyond a patient’s social networks. Some of the identified spillovers remain understudied and deserve more attention, as they could emerge across multiple disease and intervention contexts. For instance, the mental health and well-being of patients’ family members, whether or not they provide informal care, is an important area for further research [45, 47, 137, 138]. Also, reduced productivity and career-path changes, either short- or long-term, in paid and unpaid work, may be relevant for both patients and others [18, 119].

The contribution of our study is threefold. First, building on the extant literature, we propose a conceptually clear and coherent definition of spillovers that is generally applicable to economic evaluations of health interventions. It is our hope that this generic definition will reduce the current narrow association of “spillovers” with specific types of impacts on particular groups and may increase the consideration of broader impacts of health interventions. Second, the expanded inventory proposes a systematic approach for the identification of these broader consequences. The clear distinction between impacts on intervention users and potential spillover costs and effects can be adopted as is by analysts working in jurisdictions applying the societal perspective. For analysts working in jurisdictions applying a narrower perspective, like the healthcare, public sector, or insurer perspective, the definition of spillovers also provides conceptual clarity, but analysts will need to identify from the expanded inventory which impacts are relevant to consider in their specific context. Third, our work adds to existing systematic reviews of specific types of spillovers (in specific disease areas) by including a broad and extensive selection of the empirical literature on spillovers in the context of health interventions, illustrating the diversity of these impacts, demonstrating their relevance in different contexts, and highlighting gaps in order to continue improving the evidence base for intervention spillovers.

Several limitations of our study must be mentioned. First, despite our efforts to include the most relevant evidence of the variety of intervention spillovers, our search strategy was not systematic. We may have missed studies that demonstrate spillovers but do not characterise them as such, either because a different definition is used or because they are not labelled at all. Second, by proposing a definition and an expanded impact inventory, we focus on the identification stage of the process of conducting an economic evaluation. However, significant challenges related to the (proper) measurement, valuation and aggregation stages remain when including relevant spillovers. For instance, risks of double counting exist if health spillovers of caregivers are considered in addition to the “full” valuation of informal care time [6, 139], or if costs related to educational attainment are correlated with future productivity costs [19]. These issues underscore the need for practical guidance to ensure consistency and comparability in evaluations with a broader scope. Third, by relying on the impact inventory framework, our analysis of spillovers focuses on *consequences*, rather than mechanisms. Interventions aimed at promoting healthier lifestyles (e.g., smoking cessation [140]) or preventive health behaviours (e.g., vaccination uptakes [141–143]) may spill over within households through mechanisms that should be better understood. These “behavioural spillovers” [144] may result in substantial quantifiable consequences (costs and effects), especially in the long run, and thus relevant for economic evaluations and health policy design.

Fourth, given our focus on consequences, establishing connections between spillovers in the expanded inventory and elements of value in other frameworks may pose challenges. For instance, in the field of vaccinations, herd immunity and reduced antimicrobial resistance are recognised population-level benefits [145, 146]. These benefits could be included in the expanded inventory if framed as potential health spillovers—i.e., quantifiable consequences rather than mechanisms by which the health gains come about. Similarly, benefits related to health system strengthening [147] could be expressed as either cost savings in the healthcare sector or health gains for other healthcare users [53, 148]. Still, the connection to other elements of value might be less straightforward. Notably, equity concerns are increasingly discussed in economic evaluations, also in relation to spillovers and how their distribution might affect the evaluation outcome. For example, patient groups with higher care needs or larger social networks may be favoured if health spillovers are considered. Although they are typically not directly reflected in estimates of costs and effects, such considerations of normative value may and should enter the decision-making process separately and explicitly [149]. Previous authors have highlighted these equity concerns and emphasised the need of further exploration. This includes providing empirical support for these considerations (e.g., relative social value), allowing for alternative valuation strategies in secondary analyses (to maintain comparability of results with other studies and cost-effectiveness thresholds that do not consider spillovers), and disclosing any normative decisions made regarding whose impacts are considered and which weights are used (if any) [42, 51, 150].

Four important implications of our findings deserve emphasis. First, spillovers, as defined in this work, are context dependent. For instance, differences between healthcare systems (e.g., in collective coverage, use of out-of-pocket payments, and social security arrangements) will influence which costs fall on intervention users and which do not. Moreover, the relevance of different spillovers may vary between diseases and interventions. In the context of dementia care, for example, the health impacts on informal caregivers and family members may be most relevant, while for mental health interventions the costs related to the criminal justice and education sectors may be prominent, in addition to family spillovers. Nevertheless, given that spillovers are context dependent, analysts must be wary of paying selective attention to the impacts and sectors displayed in the expanded inventory. Current trends in the literature should not prevent analysts from thinking more broadly about potentially relevant spillovers in their research, such as considering “others” in a much broader sense than the direct social network of patients (e.g., potentially including colleagues, employers, taxpayers, or the general population).

Second, since our definition of spillovers is as broad as that of the societal perspective, the question of what is relevant and feasible to include in specific economic evaluation studies deserves careful attention. When applying a healthcare perspective, the set of potentially relevant spillovers typically includes health effects on others and healthcare costs falling on others. However, when applying a societal perspective, all costs and benefits are, in principle, relevant. There is no general guidance for delimiting the societal perspective in practice. Such decisions need to be made on a case-by-case basis by identifying the elements that are relevant in the context of a specific intervention, in line with the “rule of reason” proposed by the First US Panel. This includes the question of to what extent future (spillover) costs and effects need to be considered. Development of context-specific guidance, like prioritising value concepts in the field of vaccination [147], can significantly contribute to expanding the scope within a specific field in a conceptually clear and systematic way.

Third, the growing knowledge about health and health intervention spillovers is largely concentrated in higher-income settings, especially Western Europe and the United States. A consideration of spillovers in evaluations of health interventions in low- and middle-income countries (LMICs) is hampered by the lack of available secondary data [151, 152]. Differences in healthcare systems and budgets, including health insurance coverage, financial protection, and the availability of long-term care systems, may lead to considerable differences in intervention spillovers between LMICs and high-income countries. For example, the greater degree to which individuals need to bear the costs of health problems and families have to carry the burden of caring for patients highlights that spillovers can be expected to be highly relevant in LMICs as well.

Fourth, despite the growing interest in reflecting the broader value of health technologies in economic evaluations, our understanding of the magnitude of intervention spillovers is still insufficient. Namely, the magnitude of spillovers is rarely highlighted in (reviews of) evaluation studies. In our mapping review, we tried to identify applications in which spillovers were measured and statistically significant, but the latter was especially challenging. Issues arose when economic evaluations that included costs or effect measurements of others reported these measurements in aggregated format (e.g., total QALYs or total costs), thus masking the relative magnitude of specific spillovers. Moreover, most of the evaluation studies that reported spillovers were not designed to measure these impacts reliably. Typically, RCTs focus on patient outcomes and either do not measure or are not sufficiently powered or representative of actual patient populations to identify the impacts of an intervention on others than the user. Model-based studies sometimes incorporate elements like caregiver costs or utility scores derived from cross-sectional studies, which requires assumptions about the link between changes in patient health and caregiver outcomes. Robust methods to estimate caregiver outcomes from patient measurements across disease areas are a promising alternative [153], but their relations remain complex and may differ between contexts [154]. Another example of a situation where extrapolation of cross-sectional findings might provide incomplete information is the persistence of spillovers on informal caregivers after the caregiving task has ended (e.g., employment loss or bereavement). Determining caregiver outcomes using panel data would be useful in these contexts, but the number of such studies is still limited, and they rarely provide disease-specific information. More knowledge and guidance about the relevance and measurement of different types of spillovers, as well as their respective magnitudes in different contexts, will contribute to making more informed decisions about their inclusion in economic evaluations.

Hence, we propose a research agenda for spillovers, as presented in Textbox 1.

**Textbox 1**: Research agenda
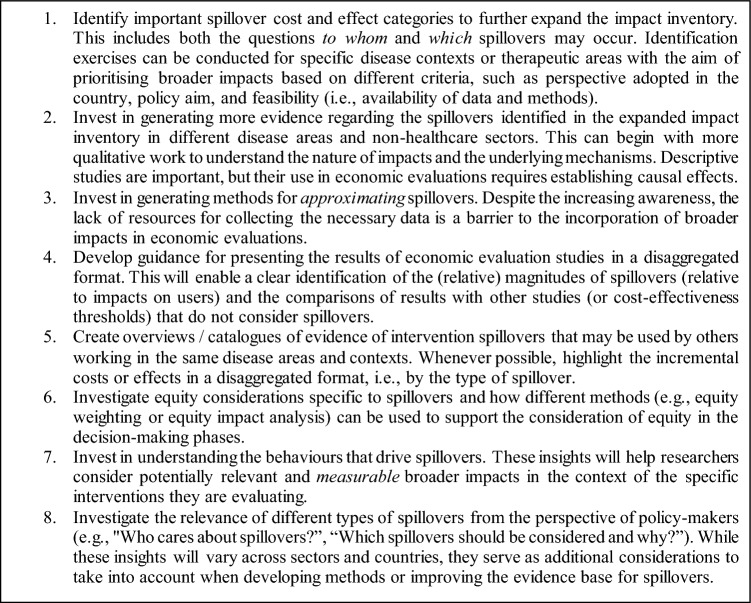


To conclude, based on a coherent definition and the documented evidence in this study, current references in the literature to the “spillovers” of health and health interventions seem to reflect only a part of the impacts outlined by the proposed definition. Conceptualising spillovers in the broadest sense, without a priori focusing on particular groups, interventions, or sectors of the economy, is a necessary step to acknowledging the full array of potential consequences of health interventions. If deemed relevant, these consequences should be measured and valued, and clearly reported. Exclusion of potentially relevant spillovers needs to be clearly justified. In light of the diversity of spillovers that can result from health interventions, our understanding of their relevance for decision-making will significantly benefit from a more consistent use of terminology, more frequent and better measurement, and increased transparency in reporting spillover costs and effects.

## Appendix 1: Search strategy

Our search strategy was implemented in two stages. The first stage identified published articles by searching the online databases PubMed and Embase in October–November 2021. We limited the start publication year to 2010 to retrieve a manageable number of studies and prioritise more recent applications and frameworks. The search query used was: (spillover* OR externalit* OR caregiver OR carer) AND health AND (economic evaluation OR societal perspective). After the identification of key reference papers and discussions among co-authors, the second stage was conducted in February–June 2022. This manual supplementary search acknowledged the use in the literature of broader terms to refer to impacts beyond the patient. Namely, “broader”, “wider”, and “non-health” in combination with “impacts”, “effects”, “benefits” and “costs”. We identified additional references via Google Scholar and snowballing (backward and forward) searches.

Titles and abstracts of the articles identified in both stages were screened for inclusion. Criteria for inclusion were (i) published in peer-reviewed journals or issued by HTA agencies, (ii) written in English, and (iii) referred to impacts in economic evaluations of patient interventions. We excluded studies analysing (i) interventions primarily aimed at improving (formal and informal) caregiver outcomes, (ii) behavioural spillovers, (iii) health policy interventions, and (iv) interventions that treated entities other than individuals, e.g., hospitals management systems. Given our interest in the concept and its application in economic evaluations, we did not restrict the search to a specific type of study. That is, we collected empirical and non-empirical works.

Works eligible for full-text assessment were classified into frameworks, conceptual analyses, commentaries or editorials, application guidelines, systematic reviews, non-systematic reviews, methodological studies and evaluation studies. While the first four categories (non-empirical studies) provided input for our conceptualisation exercise (“Spillovers: definition, implications, and identification”), the other four provided the empirical basis for the mapping review (“Spillovers in practice”). Non-empirical studies were disregarded after full assessment if no new insight about spillovers (e.g., property or scope) was discussed. With respect to empirical studies, systematic and non-systematic reviews were given priority to identify evidence of different spillover types. If available, extraction tables from reviews were used to identify studies that reported differences in spillover effects or costs across alternatives. Evaluation studies identified in the search were individually assessed. Information from reviews and evaluation studies was tabulated in a working file (1 row per study) to document the source of spillovers (intervention or illness), type of spillover (categories in Table 2, column 3), groups affected in addition to patient, measured effects or costs, and whether spillovers had been demonstrated, i.e., differences across alternatives in outcomes or costs were reported. Given that our search was not systematic, if the items considered in a particular evaluation study were similar to at least two entries in the working file, the evaluation study was no longer documented.

## Appendix 2: Additional spillover costs

### Future medical costs

Whenever spillover effects result in changes in life expectancy, future medical costs may become relevant for patients and for others, both related and unrelated to the intervention [155]. Methods for the inclusion of future medical costs have been developed, utilising estimates based on medical expenditures by age, for example [155]. In scenarios of extended patient survival, there may arise a need for additional informal care, which can cause diverse spillovers, such as medical expenditures in caregivers. Moreover, if mortality of caregivers (or family members) is affected [30], any future medical costs in added life-years would also be spillovers. In such contexts, it is important to employ in the analysis a time horizon that allows for the inclusion of all current and future medical costs of patients. Determining the relevant timeframe for also incorporating the future medical costs of others requires additional attention. Further research in this area is warranted.

### Unpaid caregiver time costs

Informal caregivers can devote a significant proportion of their time supporting patients, and it is increasingly documented that more than one individual may be providing voluntary assistance [156–159]. Time providing this support has opportunity costs; it displaces alternative time uses like paid work, unpaid work or leisure. As such, caregiving time concerns resources whose value should be reflected in economic assessments.

Available evidence of spillover caregiving time costs is typically observed in relation to diseases (rather than interventions) and usually based on cross-sectional data. For instance, comparing groups with and without caregiving duties, spillover costs in terms of caregiver time have been reported for cancer [46], stroke [160], and long-term physical injuries [117]. Using panel data, long-term consequences have also been associated with care provision. Van Houtven et al. [161] estimated reduction in work hours over time due to informal care among female caregivers of elderly adults in the United States. Similar findings have been reported for Europe [162]. A broad view on the long-term time costs associated with informal care is also necessary, as the long-term impacts can be substantial.

Health interventions may affect caregiving time directly (e.g., accompanying the patient to medical appointments) or indirectly (e.g., through changes in the patient’s health status or survival). In the context of economic evaluations, the First US Panel already acknowledged the relevance of informal caregivers’ time costs [3]. Multiple HTA guidelines also explicitly acknowledge these costs and list methodological recommendations for their measurement (e.g., Canadian [105] and Dutch [121] guidelines). Even NICE [34] in England, which adopts a narrower healthcare perspective for reference cases, recommends the quantification of these costs when the care provided might have substituted the services by the formal health and social care sectors. Building on the growing availability of methods, comprehensive guidance for the quantification, valuation and incorporation of these costs is available in the literature [6, 7, 163].

The incorporation of caregiver time costs in economic evaluations of health interventions has increased over time, which is reflected in the multiple reviews exploring this practice. Goodrich et al. [49] found that 18 of 20 economic evaluations of patient interventions included caregiver time costs, but differences across alternatives were not highlighted. More recent systematic reviews have found that the incorporation of these costs, and thus potential quantification of spillovers, is evolving differently across disease areas and patient groups. Whereas the majority of analyses included caregiver time costs in evaluations of patient-level interventions related to Alzheimer’s disease [51] and among pediatric patients [52], few studies (19 of 53) considered them in evaluations of interventions in depression [164]. Inclusion of these costs is also highlighted in other systematic reviews [165], even for different disease areas (e.g., rare diseases [166] or diabetes [167]), but the intervention user (e.g., caregiver, patient, or both) cannot be clearly distinguished from the search strategies or extraction tables.

As evidenced above, incorporating caregiver time costs in health economic evaluations is no longer rare in numbered contexts. Despite the availability of these reviews, our understanding about intervention impacts is insufficient. None of the reviews extracted information about the existence of differences in caregiver time costs across treatment arms. A closer inspection of the included studies revealed relevant examples of RCT-based and model-based evaluations. For instance, in a controlled trial of pharmacotherapy for people with dementia and depression, Romeo et al. [168] reported lower caregiving time costs for patients who received mirtazapine. Including informal care costs in the calculation of incremental costs changed the alternatives’ ranking. Conversely, Brettschneider et al. [169] did not find significant differences in caregiving time costs between patient groups who received targeted feedback upon screening for comorbid depression and those who did not. In model-based evaluations, absolute differences in caregiver time costs have been reported in favour of the intervention in the context of influenza vaccines for pediatric and elderly patients [170, 171], and medication for patients with Alzheimer’s disease [172]. Even while assessing individual evaluation studies, identifying caregiver time spillovers was further hampered by the fact that results were generally not reported in a disaggregated format. In other words, only total costs were reported, not disaggregated by cost type or separate from patient costs.

### Transportation costs

Transportation costs may spill over in different ways. For example, patient transportation costs may be borne by others (e.g., by those accompanying patients to medical appointments) or caregivers may have to travel to and from the patient’s home if they do not share a household. Transportation costs are included only occasionally in studies quantifying the burden of disease or COIs [157, 173]. In a systematic review of COIs, Mattingly et al. [110] reported that only 5 of 51 studies included caregiver transportation costs. Likewise, reviews of economic evaluations of patient interventions showed that transportation costs incurred by others are infrequently included. Lin et al. [51] found that 3 of 63 dementia-related CUAs included such costs, while Lavelle et al. [52] reported that 27 of 142 paediatric CUAs included them. Often, economic evaluations did not report these costs separately from other cost categories, with the study by Wolfs et al. [174] being a notable exception. For instance, Itzler et al. [170] reported cost spillovers, namely cost savings, from influenza vaccination on “direct non-medical costs”, which included transportation costs and care supplies. Moreover, when measured, travel costs are not always quantified separately for patients and others. This may be explained by the fact that patients and those who incur travelling expenses are often members of the same household. Some model-based studies have included caregiver travel costs based on input from experts or assumptions [175, 176].

### Legal or criminal justice

Health interventions may impact others via changes in patients’ interactions with their surroundings. In the context of certain mental health or addiction disorders, changes in criminal activity (in a broad sense) may occur, which may cause spillovers in terms of costs (e.g., through reduced damages, costs of court cases, or time from parole officers) as well as in terms of health effects (e.g., personal attacks) or well-being effects (e.g., feelings of unsafety or threats). The influence of medication on criminal behaviour has been documented in the literature. For instance, ADHD medication was associated with reductions in short term criminal rate [177]. Similarly, medication adherence was associated with fewer arrests among patients with schizophrenia spectrum disorder or bipolar disorder [178]. Janssen et al. [179] identified and validated 12 costs and benefits related to the criminal justice sector that are relevant in depression, schizophrenia, and post-traumatic stress disorder, such as police services, services in correctional facilities, pain and suffering of victims and material losses of victims or communities. Nonetheless, these impacts are generally overlooked in health economic evaluations.

The Second US Panel emphasises the relevance of identifying consequences that fall outside the healthcare sector, including the criminal justice and education sector. The Dutch and Canadian guidelines [105, 121] are among the few that recognise the potential relevance of these costs. In other regulatory frameworks (e.g., England [34]), the consideration of costs outside the health or social care sectors should be agreed upon prior the implementation of an evaluation. In a recent systematic review by Kim et al. [9], less than 5% of economic evaluations considered these consequences. Evaluation studies that included them generally considered the costs incurred in response to crime (e.g., costs of criminal justice services) and costs incurred as a consequence of crime (e.g., damage to other individuals or property). Both categories will typically constitute spillovers.

Criminal justice costs usually refer to the use of criminal justice services by individuals undergoing the intervention, which typically fall on others [125, 180]. Fewer studies have quantified the costs of the use of these services by others. For instance, in an economic evaluation of a preventive intervention for parents, Herman et al. [181] quantified outcomes of participants’ children in the long run, including the costs in the criminal justice sector. The cost savings were substantial; almost 75% of the total benefits accrued by the intervention group were cost savings related to the criminal justice sector. Spillovers in the form of costs of crimes or QoL losses of victims have also been discussed. A worked example by the Second US Panel presents a model-based evaluation of five treatment strategies for individuals with alcohol-use disorders [182], simulating lifetime legal costs for each alternative, including the tangible costs of crimes and monetarized QoL impacts on victims. Other examples include the study by Barret et al. [183] evaluating treatments for individuals with psychosis. They included criminal activity costs as well as criminal justice costs, but no statistically significant differences were identified.

### Other spillover costs

In addition to the spillovers mentioned above (and in “Spillovers in practice”), analysts have referred to other impacts by health interventions, such as housing adaptations, use of social services and changes in non-healthcare consumption. Again, the relevance of these spillovers is highly dependent on the context, e.g., housing adaptations in the evaluation of knee replacement surgery [184] and use of social services by the patient or family members in the evaluation of mental disease interventions [185]. Moreover, if alternatives under evaluation lead to different survival expectancy for patients, their future non-healthcare consumption costs become relevant if productivity gains are also considered [28]. Attention to these costs is growing but still mostly related to patients [155]. If an intervention leads to substantial longevity effects on others and their productivity gains are incorporated in the analysis, there would be grounds to evaluate the incorporation of consumption costs of others as well.

Finally, although the impact inventory list is not meant to be exhaustive, cost items that were mentioned in the literature but are not listed are direct non-medical costs and general out-of-pocket costs. These may include transportation costs, care supplies and house adaptations but also telephone bills, home meals, and any other non-medical household spending [52].

## Footnotes

1 According to recommendations of the First Panel (1996) [186], the “rule of reason applies if the cost of obtaining more precise estimates of the parameter in question would exceed the value of achieving more precision in the final cost-effectiveness result.” Two decades later, the Second Panel (2016) provided an updated characterisation: “Consequences that are expected to be trivially small in the context of the analysis, and thus to have little effect on the results, can reasonably be excluded at the analyst’s discretion.”

2 There are earlier references to “external effects”, “externalities” and “spillover effects” in the context of cost–benefit analyses of investment projects [187]. In the context of cost-utility analyses of healthcare programmes, Labelle and Hurley used the term “externalities” to refer to external benefits of healthcare consumption due to interdependent utilities and option demand [188]. Our definition of spillovers resembles that of externalities, although the latter are typically associated with the notion of unintended effects on others. Moreover, the term “spillovers” is more frequently used in the literature of health interventions [13, 32].

3 In this study, informal caregiving refers to the provision of non-professional care and support by those in patients’ social networks and is usually not compensated, although this depends on the social care system [6, 119, 189].

4 In practice, even if a country’s population is targeted, not all individuals will become users due to several reasons, e.g., lack of access to healthcare, hesitancy, or medical restrictions. Moreover, the scope of health interventions and programmes is generally confined to administrative borders. Any impacts of health programmes on non-users in neighbouring administrative areas constitute spillovers under the proposed definition.

5 The ISPOR Value Flower is another generic value framework that proposes a set of novel potential elements of value in drug assessments, such as value of hope, insurance value and scientific spillovers [190]. Empirical works documenting the feasibility of incorporating these elements are currently lacking, but exploratory studies are emerging (e.g., including insurance value [191]).

6 Pennington et al. [12] describe how the latter can have implications for the evaluation results.
